# Indentation Modulus, Indentation Work and Creep of Metals and Alloys at the Macro-Scale Level: Experimental Insights into the Use of a Primary Vickers Hardness Standard Machine

**DOI:** 10.3390/ma14112912

**Published:** 2021-05-28

**Authors:** Alessandro Schiavi, Claudio Origlia, Alessandro Germak, Andrea Prato, Gianfranco Genta

**Affiliations:** 1 INRiM—Applied Metrology and Engineering Division, National Institute of Metrological Research, Str. delle Cacce 91, 10135 Turin, Italy; c.origlia@inrim.it (C.O.); a.germak@inrim.it (A.G.); a.prato@inrim.it (A.P.); 2 Department of Management and Production Engineering, Politecnico di Torino, Corso Duca degli Abruzzi 24, 10129 Turin, Italy; gianfranco.genta@polito.it

**Keywords:** indentation hardness, indentation modulus, indentation work, Vickers hardness test

## Abstract

In this work, the experimental method and the calculation model for the determination of indentation moduli, indentation work, and indentation creep of metallic materials, by means of macroscale-level forces provided by a primary hardness standard machine at the National Institute of Metrological Research (INRIM) at the at room temperature were described. Indentation moduli were accurately determined from measurements of indentation load, displacement, contact stiffness and hardness indentation imaging and from the slope of the indentation unloading curve by applying the Doerner-Nix linear model; indentation work, representing the mechanical work spent during the force application of the indentation procedure, was determined by calculating the areas under the loading–unloading indentation curve, through fitting experimental data with a polynomial law. Measurements were performed with a pyramidal indenter (Vickers test). The applied force was provided by a deadweight machine, and the related displacement was measured by a laser interferometric system. Applied forces and the occurring indentation depths were simultaneously measured: the resulting loading–unloading indentation curve was achieved. Illustrative tests were performed on metals and alloy samples. Discussion and comments on the suitability of the proposed method and analysis were reported.

## 1. Introduction

Knowledge of elastic and plastic properties of metallic materials at the macroscopic level is of interest in many engineering and industrial applications devoted to metal processing and assembling techniques, since it can directly provide information on the material’s mechanical behavior when subjected to high stresses or for large-scale applications.

The mechanical behavior of a metallic material, for example in terms of plastic deformation and elastic recovery, cannot always be univocally identified from Hooke’s law, since certain dependences on the applied force time/rate (static, quasi-static, dynamic, and impulse), the type of acting forces (compression, tension, torsion, and penetration), the stressed surface area, the investigated scale (from the nanoscale to the macroscale level), beyond the usual environmental conditions (temperature), and the effects of aging (oxidation and corrosion) often induce some deviations from the expected linearity [1,2,3,4,5]. Therefore, it is more appropriate to identify, from time to time, a specific experimental technique tailored to the actual application of the investigated metallic material, to provide a suitable characterization of its mechanical properties in terms of elastic and plastic behavior. If it is of interest to investigate the mechanical properties of a metallic material subjected to high stresses concentrated in a single point, parameters such as indentation hardness, indentation modulus, indentation creep, and indentation work at the macroscale level allow evaluating the elastic-plastic response for typical ranges of applied stress and related deformation occurring in many practical applications.

In the metalworking industry, many assembling and processing techniques involving large stresses and punctual deformations [6,7,8,9], such as hammering [10], riveting [11], crimping [12], milling [13], cutting [14], and drilling [15], are routinely carried out. As a consequence, the accurate and reliable determination of the mechanical properties of the involved metallic materials (from working tools to materials processing) allows improving the efficiency of the whole working performance, in terms of waste reduction (metals chips), powder dispersion, lubricant consumption, machinery degradation and tools wearing, among others [16,17,18,19].

Experimental techniques based on instrumented indentation (from the nano- to macroscale level [20]) are applied to estimate elastic and plastic properties of materials. These methods, for metallic materials, are collected in ISO 14577-1 [21]. In applied metrology, “hardness” is a quantity collected within the Calibration and Measurement Capabilities of the BIPM, beyond to be supported by international key comparisons [22]. As a consequence, the elastic and plastic properties can be assessed from standardized and accurate experimental procedures, which allow guaranteeing the reliability of measurement results, based on repeatable and reproducible data.

Elastic recovery effects in indentation tests were firstly observed in 1961 by Stilwell and Tabor [23]. Back to 1983, hardness and elastic modulus, based on instrumented indentation test, were measured at the nanoscale level by Pethicai, Hutchings, and Oliver: by using a method to evaluate hardness, it was shown that depth-sensing indentation allows building a load–displacement curve, compatible with typical stress–strain diagrams of materials [24]. The method was further improved by Doerner and Nix in 1986, by using a high-resolution depth-sensing instrument [25], and in 1992, Oliver and Pharr defined a constitutive physical model of elastic indentation modulus and hardness, based on instrumented indentation [26]. It is also known that microhardness, nanohardness, and Young’s modulus strongly depend on the state of the material surface and change upon contact. In particular, electrical contact with other metals strongly depends on the physical and mechanical properties of metals as shown in [27,28]. More recently, the indentation work has been identified as a promising energy-based parameter to evaluate the elastic and plastic properties of metallic materials [29,30,31,32,33,34]. These theoretical models and experimental methods are nowadays included in ISO 14577 series; nevertheless, several important developments have been recently proposed to improve both the measurement accuracy and the reliability of empirical and semi-empirical models [35,36,37,38,39,40]. In the following, elastic and plastic properties of copper alloy, aluminum alloy, stainless steel, and copper–chromium–zirconium alloy samples are investigated, in terms of indentation modulus, indentation creep, and indentation work, at the macroscale level. Measurements are performed using a primary hardness deadweight machine, designed and realized at the National Institute of Metrological Research (INRIM), as shown in Section 2.1. In practice, by applying traditional hardness test procedures (according to ISO 6507), the elastic and plastic properties (according to ISO 14577) are evaluated at the same time, from highly accurate (and traceable) load and displacement measurements. This point is of importance, since these measurements are currently carried out by means of commercially available hardness machines in testing laboratories and industries; nevertheless, the accuracy and reliability of experimental results are often disregarded.

## 2. Materials and Methods

Materials investigated in this paper were copper alloy, aluminum alloy, stainless steel, and copper–chromium–zirconium alloy. Young’s modulus *E_s_* and Poisson ratio *ν_s_* of the tested materials were previously evaluated from accurate measurements of speeds of sound in solids and in tension at environmental temperature (~21 °C) [41,42,43,44]. Despite the known systematic difference between dynamic and static moduli, the reference data are accurate as the overall uncertainties are less than 1%. These values were determined to estimate the Poisson ratios *ν_s_* of the tested metals and alloys. In Table 1, the experimental data, used as a reference, of the tested alloys are shown.

As a reference, ISO 14577-1 [21] experimental procedures, with some specific improvements according to the literature, were implemented to evaluate the fundamental mechanical properties of metallic materials, in terms of indentation hardness *H_IT_*, indentation modulus *E_IT_*, indentation creep *C_IT_*, and the elastic part of indentation work *η_IT_*. In this section, experimental devices, test methods, and calculation models are described in detail.

### 2.1. Primary Hardness Standard Machine

**Figure 1:**
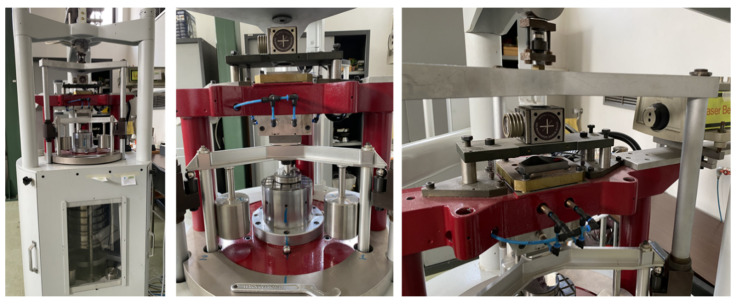
Experimental measuring systems used in this investigation: primary hardness standard deadweight machine with the details of the anvil and the interferometric system.

The indentation hardness, the indentation modulus, and the indentation work were determined by using a primary hardness standard deadweight machine from the INRIM, shown in Figure 1. The activities regarding the realization, maintenance, and improvement of the standard machine at the INRIM have been performed from the early 1970s up to the present day. Technical features and metrological characterization are summarized in detail in [45,46,47]. The standard deadweight machine complied with the requirements stated in Section 5 of the ISO 14577-1 [21]. The system applied forces by moving dead weights, and the occurring indentation depth were measured with a laser interferometric system. Applied forces were measured along the scale by a force transducer with an accuracy of 0.01%. The laser beam was aligned on the measurement axis, and the experimental data of the indentation depth were evaluated with a resolution of 0.02 µm. Force *F* and indentation depth *h* were recorded in real time at a 16 Hz sampling rate.

### 2.2. Indentation Hardness H_IT_

As stated in the standard ISO 14577-1 [21], indentation hardness *H_IT_* is determined as the ratio between the maximum load *F*_MAX_ applied and the resulting contact projected area *A_p_*, by the following equation:

(1) $$H_{IT}=\frac{F_{\mathrm{MAX}}}{A_{p}},$$

where projected contact area *A_p_*, i.e., the value of the indenter area, functioning at the contact depth, depends on depth *h_c_* of the contact of the indenter with the sample at *F*_MAX_ and on the type of the indenter. By using a Vickers diamond pyramidal indenter, with a vertex angle *α*, the projected contact area is written as:

(2) $$A_{p}={\left(2h_{c}\cdot \tan\frac{\alpha }{2}\right)}^{2},$$

where the depth *h_c_* of the contact of the indenter with the sample at *F*_MAX_ is determined from Equation (3) as follows:

(3) $$h_{c}=h_{\mathrm{MAX}}-\varepsilon \cdot \frac{F_{\mathrm{MAX}}}{S}-C_{f}\cdot F_{\mathrm{MAX}},$$

where *h*_MAX_ is the maximum indentation depth, *F*_MAX_ is the maximum of the applied force, *ε* is a quantity depending on the indenter geometry and the extent of the plastic yield in the contact (for Vickers: *ε* = 0.75), *S* is the contact stiffness, and *C_f_* is the frame compliance.

The contact stiffness *S* was determined by fitting the unloading indentation curve, as the incremental ratio *S* is expressed as: $\frac{\partial F}{\partial h_{\left|h_{\mathrm{MAX}}\right.}}$, as shown in Section 2.3.

Frame compliance *C_f_* represents the elastic deformations of the testing machine during the indentation test. Frame compliance *C_f_* was evaluated from experimental data by the slope of unloading curves, as shown in Section 2.6.

According to Annex F of the standard ISO 14577-1 [21], the values of indentation hardness *H_IT_* can be correlated to conventional Vickers hardness (HV) measured according to ISO 6507-1, [48], by applying a proper scaling factor. Divergences can be useful to identify some inaccuracies in applied procedures.

### 2.3. Indentation Modulus, E_IT_

Back to 1992, Oliver and Pharr introduced a method for the measurement of the indentation modulus based on an indentation technique [26]. Indentation modulus, *E_IT_*, quantifies the elastic response of a material subjected to the action of a concentrated load in a single point; nevertheless, the relationship between applied stress and displacements is no longer linear; thus, the indentation modulus represents a close estimation of Young’s modulus. Indentation modulus *E_IT_* depends on several parameters and boundary conditions, and according to ISO 14577-1 Annex A.5, it is expressed as [21]:

(4) $$E_{IT}=\frac{1-\nu_{s}^{2}}{\frac{2\sqrt{A_{p}}}{S\sqrt{\pi }}-\frac{1-\nu_{i}^{2}}{E_{i}}},$$

where *ν_s_* is the Poisson ratio of the sample under investigation, *ν_i_* and *E_i_* are the Poisson ratio and Young’s modulus of the indenter material, respectively, *S* is the contact stiffness, i.e., the incremental ratio between unloading force and related displacement at the maximum depth of indentation, and *A_p_* is the projected contact area.

Once the maximum applied force *F*_MAX_ and the maximum depth of indentation *h*_MAX_ were known, the indentation modulus *E_IT_* was experimentally determined from the length of the Vickers indenter diagonals and from the contact stiffness *S*, depending on the slope of the indentation unloading curve.

**Figure 2:**
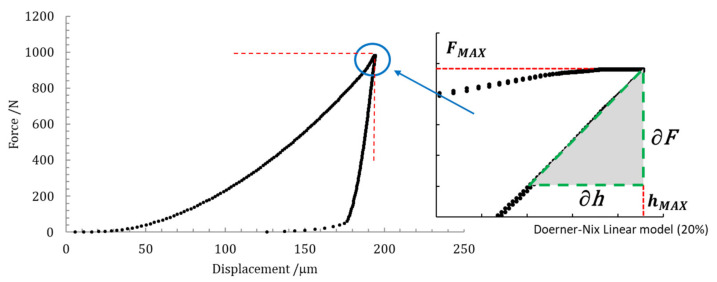
Loading–unloading paths of the indentation curve, expressed as a function of applied force and displacement, with quantities used for the determination of contact stiffness *S*.

In Figure 2, an experimental loading–unloading indentation curve as a function of true applied force and displacement and slope evaluation, is depicted.

In ISO 14577-1 [21], a linear model (the Doerner-Nix method) and a power law (the Oliver-Pharr method) are suggested to fit the unloading path of an indentation curve: the linear model is applied to the initial 20% of the unloading curve; the power-law allows fitting from 50% up to 80% of the unloading curve. However, in this paper, only the linear model was used to fit the unloading curve, since the power-law model relies on the depth of the unloading point, which has high uncertainties [38].

### 2.4. Indentation Creep

**Figure 3:**
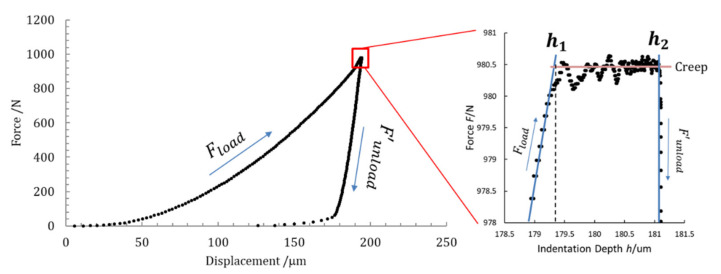
Loading–unloading paths of the indentation curve, expressed as a function of applied force and displacement with the indication of the creep of the material during the application of a constant force.

The variation of indentation depth, measured with a constant test force in a given time interval, depends on the creep of the material. Namely, indentation creep *C_IT_* is determined as the relative change of the indentation depth from the following Equation (5) according to ISO 14577-1 Annex A.6 [21]:

(5) $$C_{IT}=\frac{h_{2}-h_{1}}{h_{1}}\times 100,$$

where *h*_1_ is the indentation depth at time *t*_1_ of reaching the test force, which is kept constant and *h*_2_ is the indentation depth at the time (*t*_2_) of holding the constant test force (*h*_2_ ≡ *h*_MAX_). Different intervals of time can be applied, depending on the implemented hardness test according to ISO 6507-1 [48], usually from 10 s to 15 s. In Figure 3, the observed creep at a constant test force is shown.

### 2.5. Indentation Work

The mechanical work (i.e., the total energy) *W_total_* occurring in the indentation procedure, during the application of load and discharge, is partly dissipated as plastic deformation work *W_plast_* and partially stored as the work of the elastic reverse deformation, *W_elast_*. Thus, the mechanical work is expressed as the sum of dissipated and stored energies during the indentation processes as follows:

(6) $$W_{total}=W_{plast}+W_{elast}=\int F\cdot dh\;z,$$

**Figure 4:**
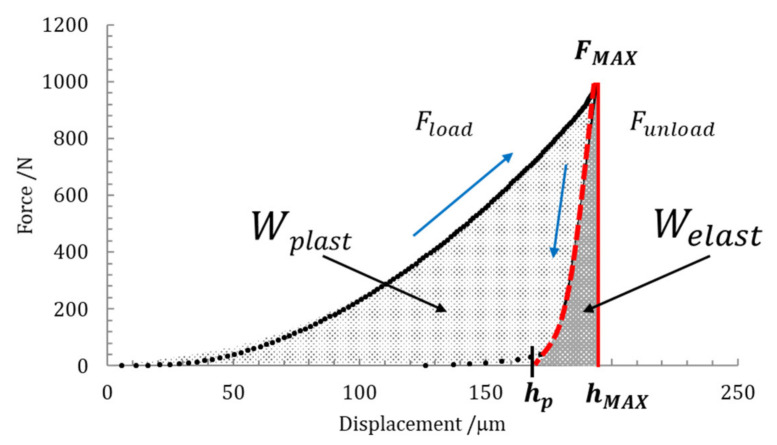
Experimental areas representing the plastic and elastic parts of the indentation work, under the loading and unloading indentation curves.

Both the plastic part and elastic part can be quantified from the calculation of the areas under the loading and unloading indentation curves. In this case, suitable curve fittings are necessary to accurately evaluate the two areas, from the experimental data diagram, as shown in Figure 4.

As shown in Figure 4, a power law is usually applied for representing the loading and unloading paths [29,30]. The general relationships are given by Equations (7) and (8), respectively:

(7) $$F_{load}=A\cdot h^{n},$$

(8) $$F_{unload}=B\cdot {\left(h-h_{p}\right)}^{m},$$

where *F_load_* and *F_unload_* are the force experimental data of the loading and unloading paths, respectively, *h* is the displacement from *F* = 0 up to *F*_MAX_ (on the loading curve) and *h_p_* is the permanent indentation depth reached from *F*_MAX_ to *F* = 0 (on the unloading curve). Empirical values of *A*, *B*, *n*, and *m* are fitting parameters, calculated from the best functions approximating the experimental data.

According to the standard ISO 14577-1 [21], the elastic part of indentation work *η_IT_* is expressed in percentage from Equation (9):

(9) $$\eta_{IT}=\frac{W_{elast}}{W_{total}}\times 100,$$

### 2.6. Frame Compliance and Contact Compliance Evaluation

Frame compliance *C_f_* is an experimental quantity affecting the accuracy of indentation modulus measurement [39], as well as the elastic–plastic deformations occurring in the sample under investigation. Frame compliance is related to the deformations of the testing machine during the indentation test. To estimate the actual frame compliance in this paper, according to ISO 14755-1 Annex C [21], a series of loading and unloading cycles, performed on a standardized stainless-steel reference block for hardness (ASAHI HV30) at a single indentation point, was applied. Actually, more procedures are available according to standards and recent literature [40,44], based on the actual indentation depth of Vickers indentation [49,50,51].

Frame compliance *C_f_* is the difference between the total compliance *C_tot_* and the sample contact compliance *C_s_* and shown as follows:

(10) $$C_{f}=C_{tot}-C_{s}={\left.\frac{\partial h}{\partial F}\right|}_{F=F_{\mathrm{MAX}}}-\frac{\sqrt{A_{p}}}{S}\frac{\sqrt{H_{IT}}}{F_{\mathrm{MAX}}},$$

where *C_tot_* is derived from the derivative of the (uncorrected) test force removal curve at the maximum force, and *C_s_* is the contact compliance of the specimen material, depending on the indentation hardness *H_IT_* (obtained with Equation (1)), the projected contact area *A_p_* (obtained with Equation (2)), and the resulting contact stiffness *S* at maximum indentation load *F*_MAX_.

**Figure 5:**
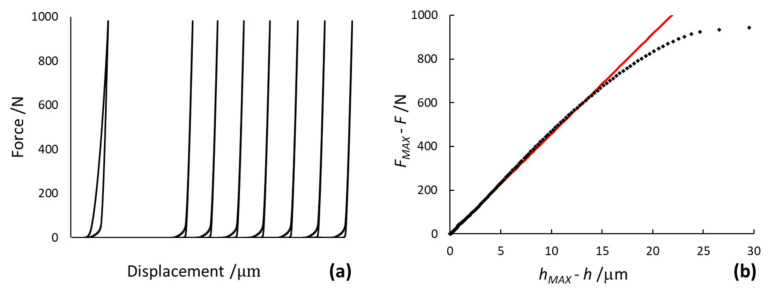
Series of loading and unloading cycles of the indentation curve at a single point (**a**); and the linear regression of the last reversed unloading curve for the HV100 procedure (**b**).

Experimentally, total compliance *C_tot_* was determined as the reciprocal of contact stiffness *S*, measured after a series of loading and unloading cycles at a single point. By measuring the slope of the indentation curve, after several repetitions, the frame compliance was calculated based on a linear regression, as shown in Figure 5.

On the basis of the experimental data, the contact compliance *C_s_* was negligible with respect to the total compliance *C_tot_*; therefore, it can be simply assumed that *C_f_* ≈ *∂h*/*∂F* for the last loading and unloading indentation curves.

In this study, *C_f_* was 94·10^−9^ m/N for HV3, *C_f_* was 36·10^−9^ m/N for HV30, and *C_f_* was 22·10^−9^ m/N for HV100. Taking into account that the experimental value of the contact compliance *C_s_* of the stainless-steel reference block sample range between 10^−13^ m/N and 10^−12^ m/N, it is possible to assume that *C_f_* ≈ *C_tot_*, at the macroscale level. As a matter of fact, by applying a series of loading and unloading cycles at a single indentation point until the slope of contact stiffness *S* kept constant, the contact compliance tended to be minimized. The resulting slope of the last indentation curve (after eight cycles in this example) is only due to the machine deformations as a whole.

## 3. Experimental Methods and Procedures

**Figure 6:**
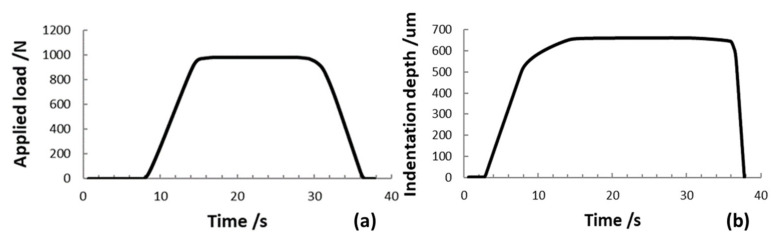
Experimental data of the applied load (**a**) and the indenter displacement (approaching and indentation) (**b**) of a HV100 indentation test. Forces were applied at a rate of 160 N/s (load displacement speed: 100 µm/s). The maximum load was applied for 15 s.

In order to define indentation modulus *E_IT_*, indentation creep *C_IT_* and indentation work *W_IT_* (and the related elastic part *η_IT_*), according to ISO 14577-1 [21] methods, HV measurements were carried out with maximum forces *F*_MAX_ of 29.4 N, 294.2 N, and 980.6 N, i.e., HV3, HV30, and HV100, according to the ISO 6507-1 procedure [48]. In the graphs of Figure 6, as an example, the experimental data of the force-controlled test procedure as a function of time, performed on the stainless-steel sample (HV100) with the INRIM primary hardness machine, are shown.

**Figure 7:**
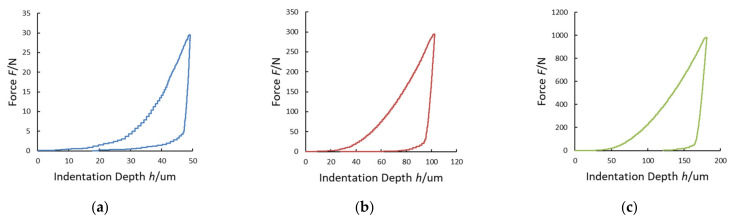
Loading–unloading curves of the Vickers hardness (HV) tests on stainless steel: (**a**) HV3; (**b**) HV30; (**c**) HV100.

**Figure 8:**
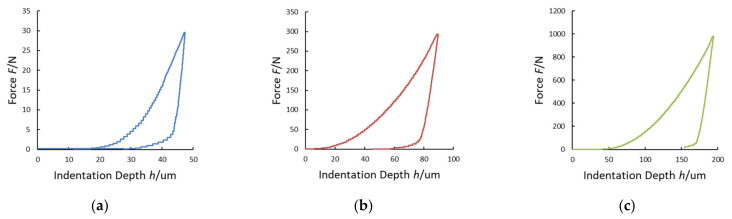
Loading–unloading curves of the HV tests on aluminum alloy: (**a**) HV3; (**b**) HV30; (**c**) HV100.

**Figure 9:**
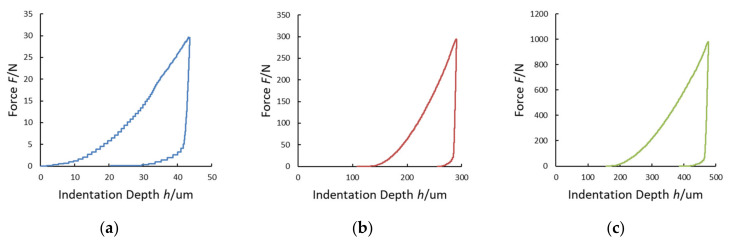
Loading-unloading curves of the HV tests on copper alloy: (**a**) HV3; (**b**) HV30; (**c**) HV100.

**Figure 10:**
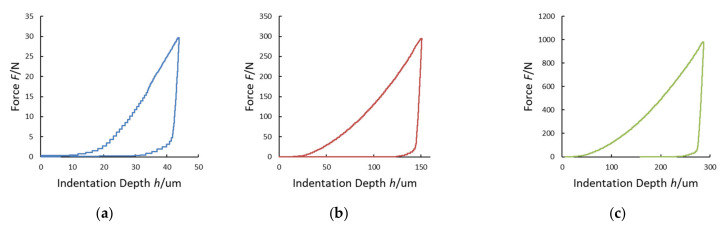
Loading–unloading curves of the HV tests on Cu–Cr–Zr alloy: (**a**) HV3; (**b**) HV30; (**c**) HV100.

The maximum indentation depth *h*_MAX_, measured by using the laser interferometric system, frame compliance *C_f_* obtained by Equation (10) and contact area *A_p_* obtained by Equation (2) were collected for every single measurement. The Young’s modulus and the Poisson ratio of the Vickers diamond pyramidal indenter are *E_i_* = 1140 GPa and *v_i_* = 0.07, respectively [21]. In the graphs of Figure 7, Figure 8, Figure 9 and Figure 10, the experimental loading–unloading curves of the HV values of the investigated metallic materials are shown.

As stated in ISO 14577-1 [21], from the ratio between maximum force *F*_MAX_ and contact area *A_p_*, as shown in Equations (1)–(3), indentation hardness *H_IT_* was determined. Parameters used to calculate *H_IT_* were applied for the determination of *E_IT_*, according to Equation (3).

Contact stiffness *S* written as *∂F*/*∂h* was determined from the Doerner-Nix linear model, by taking into account a portion of 20% of the unloading curve data, as shown in the graph of Figure 2. The geometrical dimensions of Vickers indentations, such as length *l* of the indentation side, calculated from the two diagonals *d* were accurately measured by the optical microscopy imaging technique, as described in detail in [44].

The indentation work *W_IT_* was calculated from the best fit of the loading and the unloading curves of the experimental data. As shown in Figure 4, the permanent indentation depth (at the end of the cycle) *h_p_* is the value of displacement on the unloading curve, in which the value of force is back to *F* = 0, and it was evaluated from the best fit of the experimental data. On the other hand, fitting curves introduce further uncertainties, depending on the accuracy of the mathematical model adopted. In particular, differently from the trends expressed in Equations (7) and (8), the shapes of the loading curve *F_load_* and the unloading curve *F_unload_* at the macroscale level do not show a pure exponential trend, but a more complex relationship, as can be observed in Figure 6, Figure 7, Figure 8 and Figure 9. In particular, the “knees” observed on the unloading curves (where *F →* 0) are likely due to the load-removing mechanical system of the standard machine. To avoid this effect that is material-independent, the value of *h_p_* was determined by calculating the zero value from the best fit of the unloading curve.

Moreover, since a variation of the indentation depth was achieved when the maximum constant force *F*_MAX_ is applied, as described in Section 2.4 and shown in Figure 5a, two values of the maximum indentation depth were identified, namely *h*_1_, i.e., the indentation depth at the time (*t*_1_) of reaching the test force, which is kept constant, and *h*_2_, i.e., the indentation depth at the time (*t*_2_) when the constant test force is removed (*h*_2_ ≡ *h*_MAX_). In the interval of time (*t*_2_ − *t*_1_), in which the maximum constant force *F*_MAX_ is applied, the occurring drift on indentation depth *δh* (described as: *δh* = *h*_2_ − *h*_1_) is due to the material creep, as shown in Figure 3. The mechanical work during the creep was also calculated, and the related quantity was added to the plastic deformation work *W_plast_*. As a consequence, the total mechanical indentation work *W_IT_*, given by Equation (6), was rewritten in explicit physical terms as:

(11) $$W_{IT}={\left[\int_{0}^{h_{1}}F_{load}dh+F_{\mathrm{MAX}}\int_{h_{1}}^{h_{2}}dh\right]}_{plast}-{\left[\int_{h_{2}}^{h_{p}}F_{unload}dh\right]}_{elast},$$

The whole portion of energy dissipated during loading path *h* (from 0 to *h*_1_) and the occurring creep, at maximum constant force *F*_MAX_ (from *h*_1_ to *h*_2_) is the plastic indentation work; the elastic part is quantified by the unloading path only from *h*_2_ to *h_p_*. 

**Figure 11:**
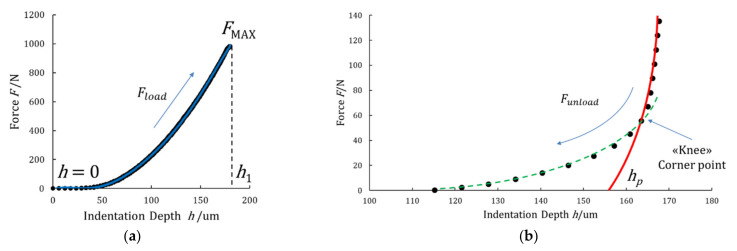
(**a**) Loading curve obtained by Equation (12); (**b**) best fit (red) of the unloading curve obtained by Equation (13) and the identification of the expected permanent indentation depth *h_p_* from the zero value of the fitting curve.

To accurately interpolate the experimental data and to build suitable (and reasonable) functions to be integrated, as shown in Equation (11), the loading and unloading curves were fitted with polynomial functions as follows:

(12) $$F_{load}=a\cdot h^{6}+b\cdot h^{5}+c\cdot h^{4}+\cdots +f\cdot h+g,\;\mathrm{for}\;0\;\leq \;h\leq \;h_{1}$$

(13) $$F_{unload}=a^{\prime }\cdot h^{5}+b^{\prime }\cdot h^{4}+\cdots +e^{\prime }\cdot h+f^{\prime },\;\mathrm{for}\;h_{p}\;\leq \;h\leq \;h_{2}$$

where the loading path *F_load_* is fitted by a single hexic-polynomial function from *h* = 0 up to *h*_1_, as shown in Figure 11a, and *F*_MAX_ is a constant during creep, as shown in Figure 3, while the unloading path *F_unload_* is fitted by a quintic-polynomial function between maximum indentation depth *h*_2_ and *h_p_* (here identified as the point in which *F* = 0 is expected on the fitting curve). The portion of the area below the unloading path, from the “knee” (identified as a corner point) back to the point at which *F* = 0 was considered within the plastic part, as shown in the graph of Figure 11b.

**Figure 12:**
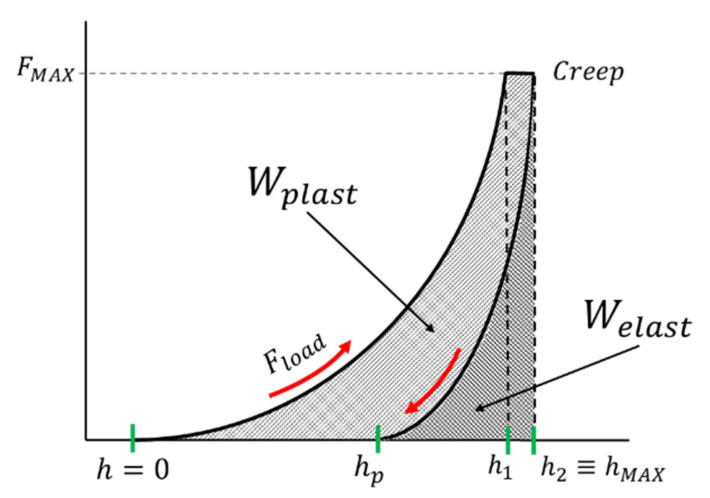
Schematic representation of the plastic, elastic, and creep parts of the indentation work beneath the loading and unloading indentation curves as a function of applied force and displacement.

In the graph of Figure 12, a representation of the whole mechanical work during the indentation procedure is schematically depicted. The part of the area under the creep drift *δh* quantifies the part of energy dissipated during the constant application of maximum load *F*_MAX_. Force application timings depend on the hardness test procedures according to pertinent standards.

## 4. Experimental Results

In Table 2, Table 3, Table 4 and Table 5, the experimental average data, and the empirical values of stainless steel, copper alloy, aluminum alloy, and copper–chromium–zirconium alloy, used to implement the calculation models of indentation hardness *H_IT_*, indentation modulus *E_IT_* indentation creep *C_IT_*, and indentation work *η_IT_* from HV standard tests, were collected. According to [52,53], plasticity characteristic *δ_H_*, which is a dimensionless parameter defined as the ratio between the plastic (*h_p_* in this specific case) and the total deformation (*h*_MAX_) during indentation, namely *δ_H_* = *h_p_*/*h*_MAX_, is also reported.

Except for HV results, of which the uncertainties are around 1% derived from BIPM-CMC [54], the uncertainty of the experimental results was evaluated by propagating the uncertainties of the experimental data listed in Table 2, Table 3, Table 4 and Table 5 including the reproducibility obtained from seven measurement repetitions on the same sample for each indentation test. The overall experimental expanded uncertainties at a confidence level of 95% were between 13% and 20%.

By way of example, the detailed uncertainty budget for indentation modulus *E_IT_* in the case of HV100 on the copper alloy sample (see Table 3) is shown in Table 6. Symbols of independent variables appearing in the mathematical model and their values and notes distinguishing the type of contribution are written down in column *x_j_*. Entries in column *u*(*x_j_*) are the standard uncertainties for each contribution. Coefficients of sensitivity *x_j_* were evaluated by partial derivation, and individual contributions *u_j_*^2^ (*E_IT_*) of the variance of dependent variable *E_IT_* were calculated. By taking into account this information, it is possible to get the expanded uncertainty *U*(*E_IT_*).

It was obtained that the major individual contribution to the combined standard uncertainty of *E_IT_* was the reproducibility obtained from seven measurement repetitions on the same sample, which can be mainly attributed to the measurement itself. In this illustrative case, the relative standard deviation due to reproducibility was around 7.8%. The standard uncertainty of experimental data was much lower than reproducibility and was 0.6%. Finally, an overall relative expanded uncertainty of 17.4% was obtained.

## 5. Discussion

The experimental results shown in Table 2, Table 3, Table 4 and Table 5, obtained according to ISO 14577-1 [21] procedures by using the primary-hardness-standard machine at the INRIM in the macroforce range, were expressed only in terms of average values. A detailed procedure for the overall uncertainty evaluation, at the macro-scale level, is available in [40]. In this paper, the indentation of materials performed according to ISO 6507-1 [48] HV tests was not limited to the HV value determination, but it was investigated by monitoring the complete cycle of the load and the unload of the test force and the occurring displacement during plastic and elastic deformation [55]. Four metallic materials, namely stainless steel, copper alloy, aluminum alloy, and copper–chromium–zirconium alloy, were subjected to HV3, HV30, and HV100 tests, according to methods routinely used at the INRIM for international key comparisons; thus, experimental HV results were determined from well-established and reproducible procedures. The contribution of the frame compliance was identified as the reciprocal of contact stiffness *S* and measured after a series of loading and unloading cycles at a single point, until the slope of contact stiffness *S* was constant and the contact compliance was minimized; frame compliance is a sensitive parameter, affecting the accuracy of contact area *A_p_* value.

Based on previous observations, once measurements are performed, a first comparison between HV values and *H_IT_* values allows identifying the trustworthiness of experimental data to be used in the calculation model: as a matter of fact, if HV ≈ *c × g*^−1^ × *H_IT_* (where *c* is the ratio of contact areas and *g* is the acceleration due to gravity and equals to approximately 1, according to ISO 14577-1 Annex F [21]), a good agreement between pure geometrical approximations of the indention and projected contact area *A_p_* is achieved; on the contrary, if large divergences are observed, such as in stainless steel (see Table 2) or in HV3 and HV100 in aluminum alloy (Table 4), data might not be reliable enough to be implemented in the calculation model, and then inaccurate determinations of the indentation modulus could be easily achieved. Compatibility between HV and *H_IT_*, observed for HV3 and HV100 in copper alloy (Table 3), HV30 in aluminum alloy (Table 4), and HV30 and HV100 in copper–chromium–zirconium alloy (Table 5), suggests consequent reliability in the inherent mechanical properties of the tested materials. It is also shown that the hardness values vary as a function of the applied load. Such behavior is likely due to size effect phenomena occurring in nano- and microhardness, rather than pile-up or sink-in effects which are negligible for HV, as shown in [40].

Nevertheless, other parameters can be used to evaluate the reliability of the experimental results by calculating the relationship between the energy dissipated during the indentation and the ratio of hardness *H_IT_* to reduced elastic modulus *E_r_* [56] and from the plasticity characteristic values *δ_H_* [52]. As observed, this energy ratio allows determining the hardness, elastic modulus, and contact area and circumventing the effects of pile-up and sink-in. The relationship is expressed as:

(14) $$\frac{W_{elast}}{W_{total}}=k^{-1}\frac{H_{IT}}{E_{r}},$$

where *H_IT_* is the indentation hardness, *E_r_* is the reduced modulus, namely *E_r_* = $\sqrt{\pi }\cdot 2C_{S}{\sqrt{A_{P}}}^{-1}$, and parameter *k*^−1^ depends on the type and on the geometry of indenter. As suggested, on the basis of FEM simulation [57], it is expected a parameter of proportionality *k*^−1^ for a Vickers indenter is five. Experimental results, obtained at macroscale force levels by applying the procedure here described, are in agreement with the proportionality of Equation (14), with an observed parameter of proportionality *k*^−1^ ≈ 7.

As previously stated, plasticity characteristic values *δ_H_* are also reported. Experimental values change as a function of the tested material, ranging, as an average, between 0.85 and 0.94 for metals and alloys as found in [52,53], and are constants as a function of the applied load. Theoretical values of *δ_H_* were also calculated by combining the Poisson’s ratio *ν_s_*, Young’s modulus *E_s_* (Table 1), and Vickers hardness *HV*, as following:

(15) $$\delta_{H}=1-14.3\left(1-\nu_{s}-2\nu_{s}^{2}\right)\frac{HV}{E_{s}}.$$

It was found that theoretical values of *δ_H_* obtained by Equation (15) are comparable with experimental ones (i.e., *δ_H_* = *h_p_*/*h*_MAX_), with mean relative differences of 2.1% for stainless steel alloy, 1.1% for aluminum alloy, 4.4% for copper alloy, and 2.6% for copper–chromium–zirconium alloy.

Although the methods for the evaluation of indentation modulus *E_IT_* are widely used, particularly at the micro- and nanoscale levels, and experimental results are often in agreement with the corresponding Young’s modulus, which is not always true at the macroscale level, as can be observed from the experimental results in Table 1 and in [43,44]. At the macro-scale level, the procedure for the calculation of the indentation work is presumably more suitable to properly evaluate the mechanical properties of tested materials, since it was founded with more reliable experimental results that it is independent of contact area *A_p_* and is based on a large set of available experimental data; as a consequence, an accurate evaluation of dissipated and stored elastic energy can be useful (beyond the reliability and representativity of large deformations due to high locally applied stresses) to estimate the actual mechanical properties of tested materials, in terms of elastic and plastic behavior and in terms of creep.

## 6. Conclusions

In this paper, the experimental procedure and the calculation models for the determination of the indentation modulus, indentation work and indentation creep of metallic materials, by applying a macro-range of force with a primary hardness standard machine at the INRIM at room temperature are presented. The experimental procedure was performed according to the standard ISO 14577-1, applied to the HV tests from HV3 up to HV100, performed on the basis of the standard ISO 6507-1. Experimental data, suitable for HV values determination, were used for the calculation of indentation hardness *H_IT_* and indentation modulus *E_IT_*; indentation work *W_IT_* was determined by calculating the areas under the loading–unloading indentation curve, from the best fit of experimental data based on specific polynomial functions; the elastic part and the plastic part (with the work due to creep) of the whole indentation work were accurately identified in terms of stored and dissipated energy. The reliability of experimental results was verified by comparing HV and *H_IT_* according to ISO 14577-1 Annex F, with the parameter of proportionality *k*^−1^ and the plasticity characteristic values *δ_H_*. From this experimental evidence, it is possible to plan suitable operations to improve the efficiency of assembling and processing techniques in the metalworking industry by reducing effects of wearing and degradation of working tools and processed metals.

## Figures and Tables

**Table 1:** Experimental values of the Young’s moduli and Poisson ratios of metals and alloys under investigation.

Material	Young’s Modulus *E_s_* (GPa)	Poisson Ratio *ν_s_ (-*)
Stainless steel	201.7	0.287
Aluminum alloy	71.6	0.342
Copper alloy	122.6	0.358
Cu–Cr–Zr	135.0	0.310

**Table 2:** Data of the HV test results on stainless steel.

Experimental Data		HV3	HV30	HV100
Maximum applied load	*F*_MAX_ (N)	29.4	294.2	980.6
Maximum indentation depth ^1^	*h*_MAX_ (µm)	49.2	102.5	181.1
Contact depth	*h_c_ (*µm)	48.0	97.9	171.4
Permanent indentation depth	*h_p_* (µm)	44.9	91.6	160.5
Maximum indentation depth 1	*h*_1_ (µm)	48.6	101.4	179.4
Creep drift	*Δh* (µm)	0.6	1.1	1.6
Contact area	*A_p_* (m)	5.64 × 10^−8^	2.35 × 10^−7^	7.20 × 10^−7^
Frame compliance	*C_f_* (mN^−1^)	9.44 × 10^−8^	3.58 × 10^−8^	2.25 × 10^−8^
Contact stiffness	*S* (Nm^−1^)	1.80 × 10^7^	4.75 × 10^7^	7.56 × 10^7^
Contact compliance	*C_s_* (mN^−1^)	5.7 × 10^−12^	5.1 × 10^−13^	1.5 × 10^−13^
**Experimental Results**				
Hardness (Vickers)	*HV*	210.8	177.5	173.9
Indentation hardness	*H_IT_* (MPa)	1297.8	1039.8	1364.3
Indentation modulus	*E_IT_* (GPa)	80.8	87.3	90.6
Elastic part of indentation work	*η_IT_* (%)	20.8	14.8	14.4
Indentation creep	*C_IT_* (%)	1.2	1.1	0.9
Plastic deformation work ^2^	*W_plast_* (µJ)	282	7533	45,760
Work of creep	*W_creep_* (µJ)	17	334	1603
Elastic deformation work	*W_elast_* (µJ)	32	1031	6923
Plasticity characteristic	*δ_H_* (-)	0.92	0.90	0.89

^1^*h*_MAX_ ≡ *h*_2_. ^2^ The plastic deformation work was computed with the work due to creep.

**Table 3:** Data of the HV test results on copper alloy.

Experimental Data		HV3	HV30	HV100
Maximum applied load	*F*_MAX_ (N)	29.4	294.2	980.6
Maximum indentation depth ^1^	*h*_MAX_ (µm)	43.6	182.3	320.4
Contact depth	*h_c_* (µm)	42.6	178.7	312.4
Permanent indentation depth	*h_p_* (µm)	38.8	173.2	302.2
Maximum indentation depth 1	*h*_1_ (µm)	43.3	181.3	318.7
Creep drift	*Δh* (µm)	0.3	1.0	1.7
Contact area	*A_p_* (m)	4.44 × 10^−8^	7.83 × 10^−7^	2.39 × 10^−6^
Frame compliance	*C_f_* (mN^−1^)	9.44 × 10^−8^	3.58 × 10^−8^	2.25 × 10^−8^
Contact stiffness	*S* (Nm^−1^)	2.15 × 10^7^	6.15 × 10^7^	9.14 × 10^7^
Contact compliance	*C_s_* (mN^−1^)	2.7 × 10^−12^	2.9 × 10^−13^	1.2 × 10^−13^
**Experimental Results**				
Hardness (Vickers)	*HV*	65.4	49.4	44.2
Indentation hardness	*H_IT_* (MPa)	662.6	521.9	410.2
Indentation modulus	*E_IT_* (GPa)	92.4	73.1	51.8
Elastic part of indentation work	*η_IT_* (%)	11.6	6.1	6.4
Indentation creep	*C_IT_* (%)	0.7	0.5	0.5
Plastic deformation work ^2^	*W_plast_* (µJ)	393	15,666	102,290
Work of creep	*W_creep_* (µJ)	9	284	1696
Elastic deformation work	*W_elast_* (µJ)	28	797	5776
Plasticity characteristic	*δ_H_* (-)	0.90	0.96	0.95

^1^*h*_MAX_ ≡ *h*_2_. ^2^ The plastic deformation work was computed with the work due to creep.

**Table 4:** Data of the HV test results on aluminum alloy.

Experimental Data		HV3	HV30	HV100
Maximum applied load	*F*_MAX_ (N)	29.4	294.2	980.6
Maximum indentation depth ^1^	*h*_MAX_ (µm)	47.4	89.6	194.1
Contact depth	*h_c_* (µm)	45.0	81.9	178.6
Permanent indentation depth	*h_p_* (µm)	41.8	72.2	161.1
Maximum indentation depth 1	*h*_1_ (µm)	47.1	89.1	193.4
Creep drift	*Δh* (µm)	0.3	0.5	0.8
Contact area	*A_p_* (m)	4.97 × 10^−8^	1.64 × 10^−7^	7.82 × 10^−7^
Frame compliance	*C_f_* (mN^−1^)	9.44 × 10^−8^	3.58 × 10^−8^	2.25 × 10^−8^
Contact stiffness	*S* (Nm^−1^)	9.32 × 10^6^	2.86 × 10^7^	4.75 × 10^7^
Contact compliance	*C_s_* (mN^−1^)	7.1 × 10^−12^	6.4 × 10^−13^	2.4 × 10^−13^
**Experimental Results**				
Hardness (Vickers)	*HV*	176.6	174.1	170.9
Indentation hardness	*H_IT_* (MPa)	1362.9	1790.5	1348.8
Indentation modulus	*E_IT_* (GPa)	58.1	67.8	52.7
Elastic part of indentation work	*η_IT_* (%)	21.4	23.8	21.8
Indentation creep	*C_IT_* (%)	0.7	0.5	0.4
Plastic deformation work^2^	*W_plast_* (µJ)	257	6325	40,914
Work of creep	*W_creep_* (µJ)	9	135	750
Elastic deformation work	*W_elast_* (µJ)	53	1704	10,573
Plasticity characteristic	*δ_H_* (-)	0.89	0.81	0.83

^1^*h*_MAX_ ≡ *h*_2_. ^2^ The plastic deformation work was computed with the work due to creep.

**Table 5:** Data of the HV test results on copper–chromium–zirconium alloy.

Experimental Data		HV3	HV30	HV100
Maximum applied load	*F*_MAX_ (N)	29.4	294.2	980.6
Maximum indentation depth ^1^	*h*_MAX_ (µm)	43.9	150.7	287.1
Contact depth	*h_c_* (µm)	42.7	146.4	278.2
Permanent indentation depth	*h_p_* (µm)	41.1	137.7	270.0
Maximum indentation depth 1	*h*_1_ (µm)	43.5	149.6	285.0
Creep drift	*Δh* (µm)	0.4	1.1	2.1
Contact area	*A_p_* (m)	4.47 × 10^−8^	5.25 × 10^−7^	1.90 × 10^−6^
Frame compliance	*C_f_* (mN^−1^)	9.44 × 10^−8^	3.58 × 10^−8^	2.25 × 10^−8^
Contact stiffness	*S* (Nm^−1^)	1.87 × 10^7^	5.18 × 10^7^	8.23 × 10^7^
Contact compliance	*C_s_* (mN^−1^)	3.1 × 10^−12^	3.5 × 10^−13^	1.2 × 10^−13^
**Experimental Results**				
Hardness (Vickers)	*HV*	90.0	62.7	57.8
Indentation Hardness	*H_IT_* (MPa)	887.8	633.0	631.5
Indentation Modulus	*E_IT_* (GPa)	91.5	70.6	91.2
Elastic part of indentation work	*η_IT_* (%)	13.5	7.6	7.3
Indentation Creep	*C_IT_* (%)	0.9	0.7	0.7
Plastic deformation work ^2^	*W_plast_* (µJ)	345	13,560	88,507
Work of creep	*W_creep_* (µJ)	11	329	2032
Elastic deformation work	*W_elast_* (µJ)	32	936	6066
Plasticity characteristic	*δ_H_* (-)	0.95	0.92	0.95

^1^*h*_MAX_ ≡ *h*_2_. ^2^ The plastic deformation work was computed with the work due to creep.

**Table 6:** Uncertainties for indentation modulus *E_IT_* of copper alloy for HV100.

Variable	Symbol	Value	Note	*u*^2^ (*x_j_*)	*c_j_*	*u_j_*^2^ (*E_IT_*)
Young’s modulus (*i*)	*E_i_* (Pa)	1.14 × 10^6^	Tabulated	negligible		
Poisson ratio (*i*)	*ν_I_* (-)	7.00 × 10^−2^	Tabulated	negligible		
Poisson ratio (*s*)	*ν_s_* (-)	3.30 × 10^−1^	Tabulated	4.8 × 10^−5^	−3.8 × 10^10^	7.0 × 10^16^
Vertex angle	*A* (rad)	2.37	Certificate	6.5 × 10^−8^	−7.4 × 10^10^	3.5 × 10^14^
Max. indentation depth	*h*_MAX_ (m)	3.20 × 10^−4^	Resolution	3.3 × 10^−17^	−1.8 × 10^14^	1.1 × 10^12^
Maximum applied load	*F*_MAX_ (N)	9.81 × 10^2^	Resolution	2.4 × 10^−3^	5.6 × 10^6^	7.5 × 10^10^
Contact stiffness	*S* (Nm^−1^)	9.14 × 10^7^	Regression	7.5 × 10^10^	5.5 × 10^2^	2.3 × 10^16^
Frame compliance	*C_f_* (mN^−1^)	2.25 × 10^−8^	Regression	1.3 × 10^−20^	1.8 × 10^17^	4.0 × 10^14^
	*E_IT_* (Pa)	4.10 × 10^9^	Reproducibility	1.6 × 10^19^	1	1.6 × 10^19^
Indentation modulus	*E_IT_* (Pa)	51.8 × 10^9^			*U*(*E_IT_*)	8.9 × 10^9^
					%	17.4

## Data Availability

The data presented in this study are available on request from the corresponding author.
